# Maternal Low-Protein Diet Deregulates DNA Repair and DNA Replication Pathways in Female Offspring Mammary Gland Leading to Increased Chemically Induced Rat Carcinogenesis in Adulthood

**DOI:** 10.3389/fcell.2021.756616

**Published:** 2022-02-01

**Authors:** Joyce R. Zapaterini, Antonio R. B. Fonseca, Lucas T. Bidinotto, Ketlin T. Colombelli, André L. D. Rossi, Laura Kass, Luis A. Justulin, Luis F. Barbisan

**Affiliations:** ^1^ Department of Structural and Functional Biology, Institute of Biosciences of Botucatu, São Paulo State University (UNESP), Botucatu, Brazil; ^2^ Molecular Oncology Research Center, Barretos Cancer Hospital, Botucatu, Brazil; ^3^ Barretos School of Health Sciences, Dr. Paulo Prata—FACISB, Barretos, Brazil; ^4^ São Paulo State University (UNESP), Itapeva, Brazil; ^5^ Instituto de Salud y Ambiente del Litoral (UNL-CONICET), Facultad de Bioquímica y Ciencias Biológicas, Universidad Nacional del Litoral, Santa Fe, Argentina

**Keywords:** perinatal programming, maternal low protein intake, DNA repair and replication, DNA damage, risk for mammary carcinogenesis, *N*-methyl-*N*-nitrosourea, female Sprague–Dawley

## Abstract

Studies have shown that maternal malnutrition, especially a low-protein diet (LPD), plays a key role in the developmental mechanisms underlying mammary cancer programming in female offspring. However, the molecular pathways associated with this higher susceptibility are still poorly understood. Thus, this study investigated the adverse effects of gestational and lactational low protein intake on gene expression of key pathways involved in mammary tumor initiation after a single dose of *N*-methyl-*N*-nitrosourea (MNU) in female offspring rats. Pregnant Sprague–Dawley rats were fed a normal-protein diet (NPD) (17% protein) or LPD (6% protein) from gestational day 1 to postnatal day (PND) 21. After weaning (PND 21), female offspring (n = 5, each diet) were euthanized for histological analysis or received NPD (n = 56 each diet). At PND 28 or 35, female offspring received a single dose of MNU (25 mg/kg body weight) (n = 28 each diet/timepoint). After 24 h, some females (n = 10 each diet/timepoint) were euthanized for histological, immunohistochemical, and molecular analyses at PDN 29 or 36. The remaining animals (n = 18 each diet/timepoint) were euthanized when tumors reached ≥2 cm or at PND 250. Besides the mammary gland development delay observed in LPD 21 and 28 groups, the gene expression profile demonstrated that maternal LPD deregulated 21 genes related to DNA repair and DNA replication pathways in the mammary gland of LPD 35 group after MNU. We further confirmed an increased γ-H2AX (DNA damage biomarker) and in ER-α immunoreactivity in mammary epithelial cells in the LPD group at PND 36. Furthermore, these early postnatal events were followed by significantly higher mammary carcinogenesis susceptibility in offspring at adulthood. Thus, the results indicate that maternal LPD influenced the programming of chemically induced mammary carcinogenesis in female offspring through increase in DNA damage and deregulation of DNA repair and DNA replication pathways. Also, *Cidea* upregulation gene in the LPD 35 group may suggest that maternal LPD could deregulate genes possibly leading to increased risk of mammary cancer development and/or poor prognosis. These findings increase the body of evidence of early-transcriptional mammary gland changes influenced by maternal LPD, resulting in differential response to breast tumor initiation and susceptibility and may raise discussions about lifelong prevention of breast cancer risk.

## Introduction

Breast cancer is one of the most common malignancy in women worldwide and the second leading cause of cancer deaths among women (Harbeck and Gnant, 2017; Ferlay et al., 2018). The well-established risk factors for breast cancer development include age, inherited genetic mutation, hormone replacement, nutritional deficiency, lifestyle, and environmental factors (Sun et al., 2017; De Cicco et al., 2019; Fahad Ullah, 2019).

Studies have shown that breast cancer susceptibility might be predetermined because of intrauterine/neonatal programming (Hilakivi-Clarke and de Assis, 2006; Fernandez-Twinn et al., 2007; Fernandez-Twinn and Ozanne, 2010; Beinder et al., 2014; da Cruz et al., 2018). Fetal programming occurs during embryonic and fetal development, a critical period in which tissues and organs are formed, and refers to the heritable changes in gene expression that can influence diseases later in life (Barker et al., 2002; Barker, 2007). Therefore, stimulus or insult at this critical period can result in developmental adaptations that produce structural, physiological, and metabolic changes, thereby predisposing descendants to chronic diseases in adulthood, including cardiovascular and metabolic diseases and cancer (Barker et al., 2002; Kwon and Kim, 2017; Herring et al., 2018). Human and animal data have shown that maternal postconception malnutrition, especially low dietary protein intake, can cause embryonic losses and intrauterine growth restriction (IUGR) that leads to hormone imbalances, metabolic disorders, and cell signaling defects (Fernandez-Twinn and Ozanne, 2010; Wu et al., 2012). In the meantime, these alterations have been associated with increased breast cancer risk (Mellemkjær et al., 2003; Ozanne et al., 2004; Diaz-Santana et al., 2020). Hence, the perinatal alterations induced by maternal low-protein diet (LPD) intake can increase the susceptibility of the epithelial mammary cells to tumor initiation induced by environmental carcinogens (Fernandez-Twinn et al., 2007; Fernandez-Twinn and Ozanne, 2010; Diaz-Santana et al., 2020).

The use of animal models to study fetal programming elucidates the relationship between maternal environment and offspring’s health (Hilakivi-Clarke and de Assis, 2006). Besides, the chemically induced carcinogenesis model is an important tool to study the multistep process of mammary carcinogenesis (Russo and Russo, 1996; Russo, 2015). Using a maternal LPD model, our research group and others reported important changes in several organs, such as liver, mammary gland, pancreas, prostate, and adipose tissue (Plank et al., 2006; Fernandez-Twinn et al., 2007; Beinder et al., 2014; Santos et al., 2019; Varuzza et al., 2019; Alejandro et al., 2020; de Oliveira Lira et al., 2020). Fernandez-Twinn et al. (2007) were the first to demonstrate the adverse effects of gestational and lactational LPD on *N*-methyl-*N*-nitrosourea (MNU)–induced mammary carcinogenesis model in female offspring from Wistar rats, using a total of three 50 mg/kg body weight (b.w.) injections at 3, 4, and 5 weeks of age in a resistant rat strain. In that pioneer study, the maternal LPD resulted in female offspring with low birth weight, increased insulin-like growth factor 1 (IGF-1) and estrogen expression, and reduced postnatal ductal branching and epithelial invasion followed by compensatory mammary growth. In addition, the maternal LPD had long-term effects in offspring adulthood including the development of hyperinsulinemia, insulin resistance, diabetes mellitus type 2, and increased risk of early-onset mammary tumorigenesis induced by MNU (Fernandez-Twinn et al., 2007). In absence of carcinogen administration, most molecular findings in female offspring mammary gland were fed a gestational LPD addressed transcriptional alterations toward cell cycle control, insulin resistance, and reactive oxygen species (ROS) pathways (Zheng et al., 2012; Beinder et al., 2014). Beinder et al. (2014) observed impairment in mammary gland development in female offspring from the Wistar rats whose dams were fed an LPD, as well as identified differential regulation of genes and pathways for factors regulating cell cycle and growth. Furthermore, gestational LPD modulates p21 gene expression and histone modifications within its promoter in the mammary gland of offspring rats that can predispose the female offspring rats to the risk of developing mammary cancer later in life (Zheng et al., 2012).

Hence, these findings suggest that intrauterine and early postnatal environments, such as a maternal low protein intake, play an important role for the developmental initiation of mechanisms underlying the programming of breast cancer in adulthood. In the classic Dutch-famine study, Painter et al. (2005) observed that IUGR in the first trimester of pregnancy is associated with an earlier reproduction phase postpartum, earlier onset of menopause, and risk for breast cancer in adulthood. Also, both environmental and dietary postnatal influences are as important as fetal programming itself for mammary gland development, as previously suggested (Russo and Russo, 1996; Hilakivi-Clarke and de Assis, 2006). Based on these findings, the postnatal phase seems to play a distinct role for mammary gland development following IUGR in the rat. Thus, the combination of the chemically induced mammary carcinogenesis model and the fetal and postnatal programing animal model is an important tool to study the uterine/neonatal environmental effects on cell vulnerability to malignant transformation. However, the molecular mechanisms involved in chemically induced mammary carcinogenesis susceptibility by maternal LPD intake, especially during gestation and lactation, are still poorly understood. Given that a poor maternal-protein diet is observed in both gestational and lactational phases, and the breast cancer prevention remains challenging in the world, understanding how a maternal-protein diet can drive the susceptibility to mammary tumorigenesis provides prevention strategies. Therefore, this study investigated the effects of gestational and lactational LPD on the gene expression of key pathways involved in mammary tumor initiation after 24 h of MNU administration, as well as on the breast cancer susceptibility in female offspring Sprague–Dawley rats, a susceptible rat strain that mimics the human disease (Russo, 2015). The findings presented here show that intrauterine and lactational protein restriction leads to early-transcriptional mammary changes (i.e., DNA repair and DNA replication pathways) followed by an increased incidence of mammary tumor later in life in female offspring challenged with an acute MNU dose in critical postnatal windows of mammary gland development.

## Materials and Methods

### Animal Housing and Experimental Design

All animal procedures in this study followed the Ethical Principles in Animal Experimentation adopted by the Brazilian College of Animal Experimentation (COBEA). This study received institutional approval from the Ethics Committee for Animal Use of the Bioscience Institute/UNESP (CEUA) (protocol 1106). Adult female (n = 60) and male (n = 30) Sprague–Dawley rats (90 days of age) were purchased from Multidisciplinary Center for Biological Research at the University of Campinas (UNICAMP, Campinas, São Paulo, Brazil). The animals were kept in a room under a controlled temperature (22°C–25°C), relative humidity (55%), and a photoperiod (12 h), with free access to water and food.

Virgin female rats were mated overnight with established male breeders, and the detection of spermatozoa and positive cytology for estrus phase in the vaginal smear was designated as gestational day 1 (GD1). Thus, the pregnant rats were fed a normal-protein diet (NPD) (17% protein) or an LPD (6%) from GD1 to postnatal day 21 (PND 21). Normoprotein and LPDs were provided by PragSoluções (Jaú, São Paulo, Brazil). These diets have been previously described as isocaloric and normosodic based on an AIN-93G formulation (Colombelli et al., 2017; Santos et al., 2019) (Supplementary Table S1). To maximize lactation performance, litter size was standardized to eight pups per litter (four females and four males). After weaning, the female offspring were allocated into two groups (NPD, n = 61; and LPD, n = 61) (separated cage/group) (Supplementary Figure S1). In the rat prepubertal phase, the mammary gland development shows two physiologic peaks at PND 28–29 (ductal morphogenesis) and PND 34–35 (Sinha and Tucker, 1966; Russo and Russo, 1996). Thus, female offspring received a single intraperitoneal dose of 25 mg/kg b.w. of MNU (Sigma–Aldrich, St. Louis, MO, United States) dissolved in phosphate-buffered saline (PBS) acidified with acetic acid (Thompson and Adlakha, 1991) (n = 28 per diet/timepoint) at either PND 28 or 35. After 24 h of carcinogen administration, female offspring (n = 10 per diet/timepoint, one female/litter) were euthanized. The remaining animals (n = 18 each diet/timepoint, two female/litter) were followed to analyze the tumor formation until PND 250 (maximum period) and euthanized if the tumor reached ≥2 cm before PND (Supplementary Figure S1). Some female offspring were euthanized at PND 21 (n = 5 per diet, one female/litter) to evaluate the effects of LPD on mammary gland development prior to be switched to normal-protein diet.

For female Sprague–Dawley rats, the dose range of MNU administration is 25–80 mg/kg b.w. (Russo and Russo, 1996). In our study, the animals received a dose of MNU (25 mg/kg b.w) because it results in a low number of mammary tumors. This enabled evaluating the effects of maternal protein restriction on increasing the number of mammary adenocarcinomas in offspring. All animals were euthanized by exsanguination under sodium pentobarbital anesthesia (75 mg/kg b.w.). For each euthanasia, specific analyses were performed: whole-mount mammary gland growth (PND 21; PND 29 and PND 36) and serum estrogen and progesterone, immunohistochemistry, histopathology, and gene expression (PND 29 and 36). The tumor histology was performed in all tumor samples collected. The analysis descriptions are in the following sections. All analyses were performed comparing NPD and LPD groups on the same PND.

### Blood Serum, Whole-Mount, and Immunohistochemical Mammary Gland Analyses

Blood samples (n = 5 each diet/timepoint, one female per litter) were centrifuged (2,400 *g* for 20 min), and the serum was stored at −20°C for hormonal analysis. Serum estrogen (17β-estradiol, Monobind®, 4,925-300 CA, USA; sensitivity: 6.5 pg/mL) and progesterone (Monobind®, 4,825-300, CA, USA; sensitivity: 0.105 ng/mL) were determined by colorimetric methods according to the manufacturer.

The fourth right abdominal mammary gland of female offspring (n = 5 each diet/timepoint, one female per litter) was collected and air-dried on the histological slide for 10–15 min on a clean glass slide and fixed in buffered formalin 10% for 48 h. The slides were washed in 70% ethanol, rinsed in water, and stained with carmine (1 g) and aluminum potassium sulfate dodecahydrate (2.5 g) (Sigma–Aldrich) for 4 days. Afterward, mammary gland whole mounts were dehydrated in sequential steps of ethanol (70%, 95%, and 100%), cleared in xylene, and mounted with Permount and coverslipped (Russo and Russo, 1996; Russo, 2015). Mammary gland tree was photographed using the magnifying glass at 1× magnification (Leica MZ12 DF C 420; Japan) coupled to a capture system and image analysis. Different parameters were measured for each mammary gland tree representing its outgrowth: ductal elongation, transversal growth, area, and perimeter. The total number of terminal end buds (TEBs) in the entire external margin of the mammary gland was determined as previously described (Russo and Russo, 2006) under a microscope (Olympus Bx 53F, Japan; 20× objective).

The fourth left abdominal mammary gland (n = 5 animal/diet/timepoint, one slide/animal, one female per litter) was fixed in 10% phosphate-buffered formalin for 24 h, embedded in paraffin blocks, and cut into 5-μm-thick sections, which were stained with hematoxylin–eosin (HE) or immunohistochemically for Ki-67, ER-α, and γ-H2AX. Histological sections were placed on silanized-coated slides, deparaffinized, and rehydrated with graded alcohol. These sections were subjected to Pascal pressure chamber retrieval in a citrate acid buffer at pH 6.0 at 120°C for 30 min. Endogenous peroxidase was blocked with 3% H_2_O_2_ in PBS for 10 min in the dark. After washing with PBS, slides were incubated with nonfat milk in PBS for 60 min. Sections were then incubated with rabbit monoclonal anti–Ki-67, 1:100 dilution (Abcam, United Kingdom); mouse monoclonal anti–ER-α, 1:50 dilution (Invitrogen, EUA); and anti–γ-H2AX, 1:200 dilution (Invitrogen, EUA) primary antibodies in a humidified chamber (overnight, 4°C). Then, the slides were incubated with one-step horseradish peroxidase polymer (EasyPath–Erviegas, Brazil) (20 min). The reaction was visualized with 3-diaminobenzidine chromogen (Sigma–Aldrich, USA) and counterstained with Harris hematoxylin.

Apoptosis was analyzed in HE-stained slides, using morphological criteria (Elmore et al., 2016). Ki-67, ER-α, and γ-H2AX labeling indexes (LI%), and apoptosis indexes (AI%) were calculated as the number of positively marked or apoptotic epithelial cells divided by the total number of cells scored ×100 (400–500 cells/mammary gland per animal). For all histological analyses, 25 randomly selected fields were considered.

### Mammary Tumor Analysis Until PND 250

After MNU administration at either PND 28 or 35, the remaining animals (n = 18 each diet/timepoint, two female/litter) were followed to analyze the tumor formation until PND 250 (maximum period) and euthanized if the tumor reached ≥2 cm before PND 250. Female offspring were examined three times per week to record the presence of gross mammary tumors and the number and location of each palpable mass in different mammary gland complexes. The body weight was analyzed at birth and PND 21, 28, 35, 50, 75, 100, 125, 150, 175, 200, 225, and 250 (statistical analysis was performed at each timepoint and compared between LPD groups vs. their respective NPD groups). Data of body weight gain (g) were obtained from PND 28 to 250 among the carcinogen-treated groups that received the MNU in the same PND. For histological analysis, tumor samples were collected and fixed in 10% phosphate-buffered formalin for 24 h, embedded in paraffin blocks, cut into 5-μm-thick sections, and stained with HE. Mammary lesions were classified according to the previously published criteria (Russo, 2015). Tumor incidence (percentage of animals with tumors) and tumor latency (time between MNU administration and appearance of the first palpable tumor per animal) were recorded for each group (Russo, 2015).

### Gene Expression

Twenty-four hours after MNU administration, the fourth right abdominal mammary glands from the female offspring in NPD and LPD groups (n = 5 each diet/timepoint, one female per litter) were removed and stored at −80°C. Total RNA was extracted using the RNeasy Mini kit (Qiagen, Hilden, Germany) followed by on-column DNA digestion. RNA samples were solubilized in nuclease-free water (Qiagen), and their concentration and integrity were evaluated on a NanoVue™ Plus (GE Healthcare) and an Agilent 2100 bioanalyzer (Agilent Technologies, Boeblingen, Germany), respectively. Equal quantities (20 ng/μL) of total RNA from each sample were reverse-transcribed to the first-strand cDNA using High-Capacity cDNA Reverse Transcription Master Mix (Life Technologies, EUA) according to the manufacturer’s instruction.

RNA expression profiles were compiled using 96-well TaqMan® Array Cards (TAC)-based real-time polymerase chain reaction (PCR). The custom TAC assessed 96 genes involved in cell proliferation, DNA damage, DNA repair, and apoptosis (Supplementary Data S1). Actb, Pum1, and Trfc genes were used as housekeeping genes to normalize mRNA expression. Target genes were amplified using the TaqMan® Universal Mastermix II (Life Technologies, USA) by a cycling protocol of heat activation at 50°C for 1 min and denaturation at 95°C for 10 min followed by 40 cycles of 95°C for 15 s and 60°C for 1 min. Fluorescence detection was performed on QuantStudio™ 12 K Flex Real-Time PCR System (Life Technologies). The relative expression of target genes was analyzed by the comparative Ct method (ExpressionSuite™ software; Life Technologies). This study was conducted according to the MIQE (Minimum Information for Publication of Quantitative Real-Time PCR experiments) guidelines (Bustin et al., 2009).

### Functional Enrichment Analysis

The deregulated genes were used to identify overrepresented gene ontology categories of biological processes and pathways with the Database for Annotation, Visualization, and Integrated Discovery (DAVID v6.8) (available at https://david.ncifcrf.gov/tools.jsp). The functional information was assessed using UniProtKB database (available at http://www.uniprot.org/). Protein-protein interaction (PPI) networks codified by deregulated genes were generated using Metasearch STRING (v10.5.1) and visualized by Cytoscape (v3.4.0). Nomenclature of genes was established by the Rat Genome Nomenclature Committee (https://rgd.mcw.edu/nomen/nomen.shtml).

### Characterization of Mammary Gland Molecular Markers With Breast Cancer Patients Using an *In Silico* Approach

For further translational insights into the relationship of molecular markers observed in female offspring mammary gland whose dams were fed an LPD with breast cancer prognostic prediction, the SurvExpress database (http://bioinformatica.mty.itesm.mx:8080/Biomatec/SurvivaX.jsp) was used for risk assessment in the BRCA-TCGA breast invasive carcinoma dataset. This tool allowed the association between the set of differentially expressed genes observed in LPD 35 group with the survival of patients with breast cancer using Cox proportional risk regression, according to the risk groups estimated by an optimization algorithm. First, using the 21 deregulated genes found in the LPD 35 group in comparison to the NPD 35 group, the univariate Cox analysis was performed, and we selected the genes with (*p* < 0.1) for breast cancer prognostic prediction in a multivariate analysis. In this additional analysis, we considered *p* < 0.05 as statistically significant.

### Statistical Analysis

Changes in body weight and food intake, mammary development (mammary gland outgrowth and number of TEBs), serum hormones, tumor latency, LIs (Ki-67, ER-α, and γ-H2ax), and AIs were analyzed by Student *t* test. Kaplan–Meier log-rank test was performed for comparing tumor-free animals. The percentage of different tumor phenotype (%) and tumor incidence were analyzed by *χ* ^2^ test. Statistical analysis was performed in GraphPad Prism software (version 6.01; La Jolla, CA, USA). Significant differences were assumed when *p* ≤ 0.05.

For gene expression, Student *t* test was applied to perform pairwise comparisons, considering a fold change of ≥1.5. The significant overrepresented gene ontology categories and pathways were assumed when a false discovery rate was ≤0.05. In Cox proportional risk regression, we considered *p* < 0.1 and *p* ≤ 0.05 for univariate and multivariate analyses, respectively.

## Results

### Female Offspring Body Weight Is Affected by Maternal LPD

There was no significant difference between the NPD and LPD groups regarding the number of total pups/litter and the number of male/female pups per litter (gender distribution) (data not shown). Body weight at birth (PND 1) and weaning (PND 21) and body weight evolution of groups are indicated in Table 1. In the present study, we observed that maternal low protein restriction influenced offspring body weight, which is in line with other experimental and human studies (Fernandez-Twinn et al., 2007; Herring et al., 2018; Bautista et al., 2019; Varuzza et al., 2019; Yang et al., 2020). During the experimental period (PND 1 to PND 250), the body weight of female offspring whose dams were fed an LPD was significantly lower (*p* < 0.001) than female offspring whose dams were fed with NPD. Body weight gain was measured from PND 28 to 250. There was no significant difference in body weight gain between the LPD and NPD groups after MNU tumor initiation and NPD reintroduction after weaning (NPD 28 vs. LPD 28, *p* = 0.314) (NPD 35 vs. LPD 35, *p* = 0.083) (Table 1).

**TABLE 1:** Effects of gestational and lactational low-protein diet and MNU administration on female offspring body weight evolution. ^a^

Postnatal day (PND)	Group/Treatment^b^			
	NPD		LPD	
Birth weight (g)	6.8 ± 0.6		5.78 ± 0.8^d^	
PND 21	48.4 ± 4.3		22.1 ± 4.5^d^	
	NPD 28	LPD 28	NPD 35	LPD 35
PND 28	82.8 ± 9.0	44.8 ± 8.5^d^	80.3 ± 8.1	47.8 ± 6.6^d^
PND 35	105.8 ± 6.5	73.2 ± 6.8^d^	120.5 ± 13.0	71.5 ± 10.6^d^
PND 50	177.7 ± 9.8	135.7 ± 16.2^d^	178.9 ± 14.4	142.4 ± 10.8^d^
PND 75	216.4 ± 9.6	179.9 ± 11.5^d^	231.5 ± 8.3	188.2 ± 10.1^d^
PND 100	236.8 ± 12.8	203.0 ± 14.0^d^	253.1 ± 10.0	210.9 ± 13.5^d^
PND 125	255.2 ± 15.5	216.0 ± 14.8^d^	266.1 ± 8.6	225.3 ± 15.1^d^
PND 150	265.3 ± 14.3	225.6 ± 14.2^d^	276.6 ± 7.0	233.9 ± 15.4^d^
PND 175	272.1 ± 15.9	232.9 ± 14.4^d^	283.6 ± 7.1	240.6 ± 14.4^d^
PND 200	274.1 ± 15.8	243.9 ± 13.9^d^	289.7 ± 10.0	247.1 ± 13.2^d^
PND 225	277.6 ± 16.3	246.8 ± 13.1^d^	294.9 ± 8.9	251.8 ± 12.6^d^
PND 250	284.3 ± 18.9	253.2 ± 12.7	301.3 ± 8.8	255.3 ± 16.5^d^
Body weight gain (g)^c^	205.1 ± 20.4	214.5 ± 14.0	227.5 ± 8.7	211.4 ± 16.9

a Values are mean ± standard deviation.

b Normoprotein diet (17% casein). LPD: low-protein diet (6% casein); PND, postnatal day; MNU–postnatal day initiation at 28 or 35; MNU, *N*-methyl-*N*-nitrosourea administered as a single intraperitoneal dose of 25 mg/kg at postnatal day 28 or 35.

c Difference between PND 250 and PND 28 for tumor-free animals or tumor-bearing rats with small tumors (<2 cm).

d Different from NPD, group with the same MNU–postnatal day initiation. Student *t* test (*p *< 0.001).

### Maternal LPD Induces a Delay in Female Offspring Mammary Gland Development

**FIGURE 1:**
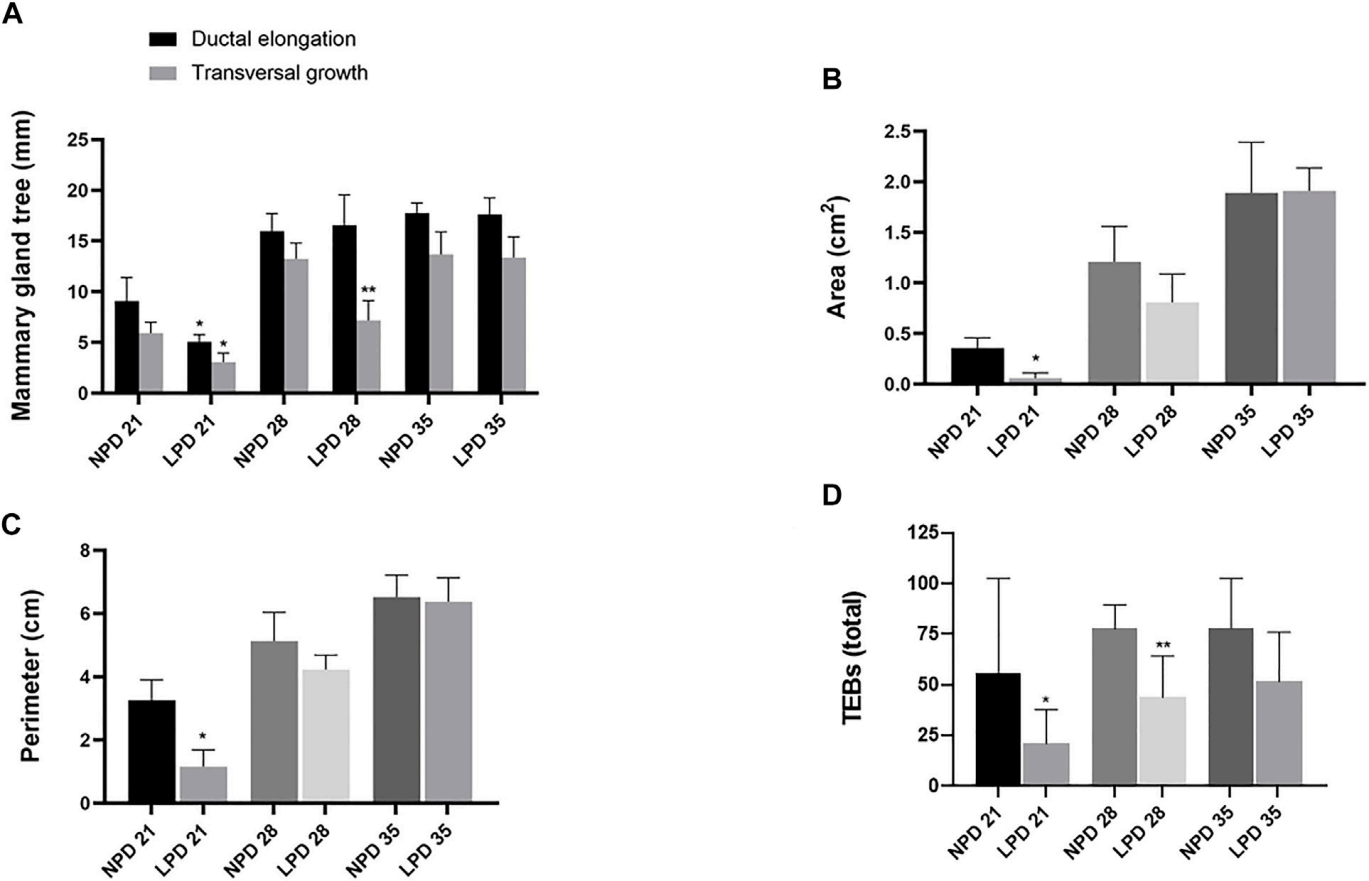
Maternal low-protein diet programs mammary gland development of female offspring rats. Mammary gland measurements: **(A)** Ductal elongation and transversal growth (mm). **(B)** Area (cm^2^). **(C)** Perimeter (cm). **(D)** The number of terminal end buds (TEBs) per field in the external margin of the mammary gland. Values expressed as mean ± standard deviation. *,**Significant different from NPD 21 and NPD 28, respectively. The differences were determined by Student *t* test (0.001 ≤ *p* ≤ 0.049). NPD: normoprotein diet. LPD, low-protein diet. Postnatal day of euthanasia (21) and Postnatal day of MNU administration (28 or 35). MNU, *N*-methyl-*N*-nitrosourea administration (25 mg/kg, i.p.; single dose).

**FIGURE 2:**
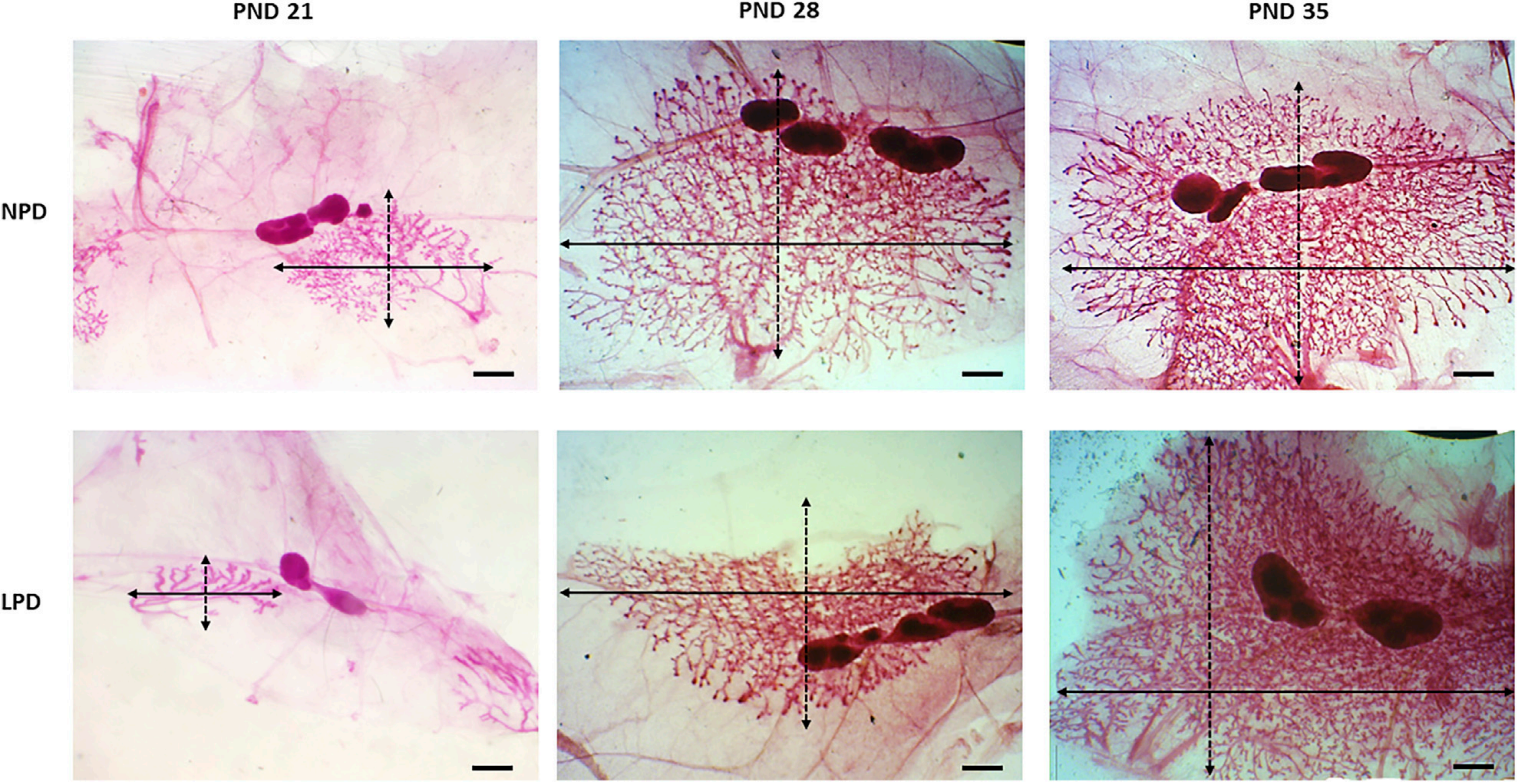
Representative images of the whole-mount-stained mammary gland. Outgrowth measurements in each group: transversal growth (black dotted arrow) and ductal elongation (black arrow). LPD, low-protein diet; PND, postnatal day (scale bar = 20 µm).

**FIGURE 3:**
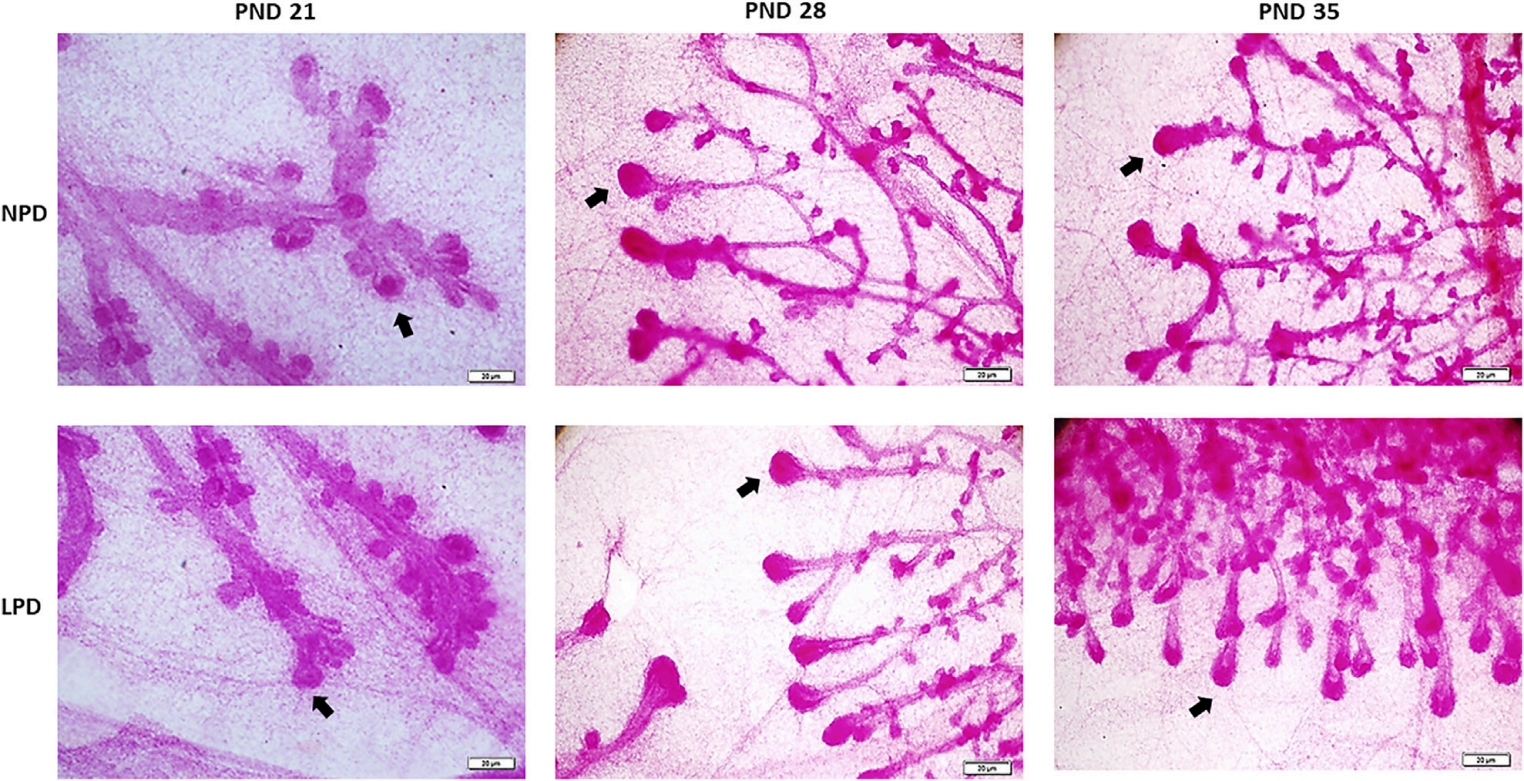
The terminal end buds (black arrows) in the external margin of the mammary gland. NPD, normoprotein diet; LPD, low-protein diet; PND, postnatal day (scale bar = 20 µm).

Aiming to investigate if maternal low protein intake could induce developmental changes in a hormone-responsive organ, we evaluated the mammary gland development through whole-mount preparations. The morphometric analysis of mammary gland outgrowth and the number of TEBs are shown in Figure 1. In addition, representative images of whole mounts prepared from each group are shown in Figure 2 and Figure 3. A significant reduction in ductal elongation and transversal growth (*p* ≤ 0.001 and *p* ≤ 0.0023, respectively) of the abdominal mammary gland in LPD 21 and a significant reduction (*p* ≤ 0.0023) in transversal growth of LPD 28 were observed in comparison to their respective NPD groups at these timepoints (Figure 1A, and Figure 2). Besides, the mammary gland area and perimeter were also significantly lower (*p* < 0.001) in LPD 21 (Figures 1B,C). At PND 35, all these mammary growth parameters were similar between the NPD 35 and LPD 35 groups, demonstrating the catch-up mammary growth after feeding with adequate-protein diet (Figure 2). Following the mammary gland development delay observed at PND 21 and PND 28 in the LPD groups, the number of the TEBs was lower in LPD 21 (*p* = 0.049) and LPD 28 (*p* = 0.010) (Figure 1D, and Figure 3). There was no significant difference in estradiol and progesterone serum levels between the NPD and LPD groups (*p* > 0.05) (Supplementary Figure S2).

### Mammary Tumor Susceptibility Is Increased by Maternal LPD

**FIGURE 4:**
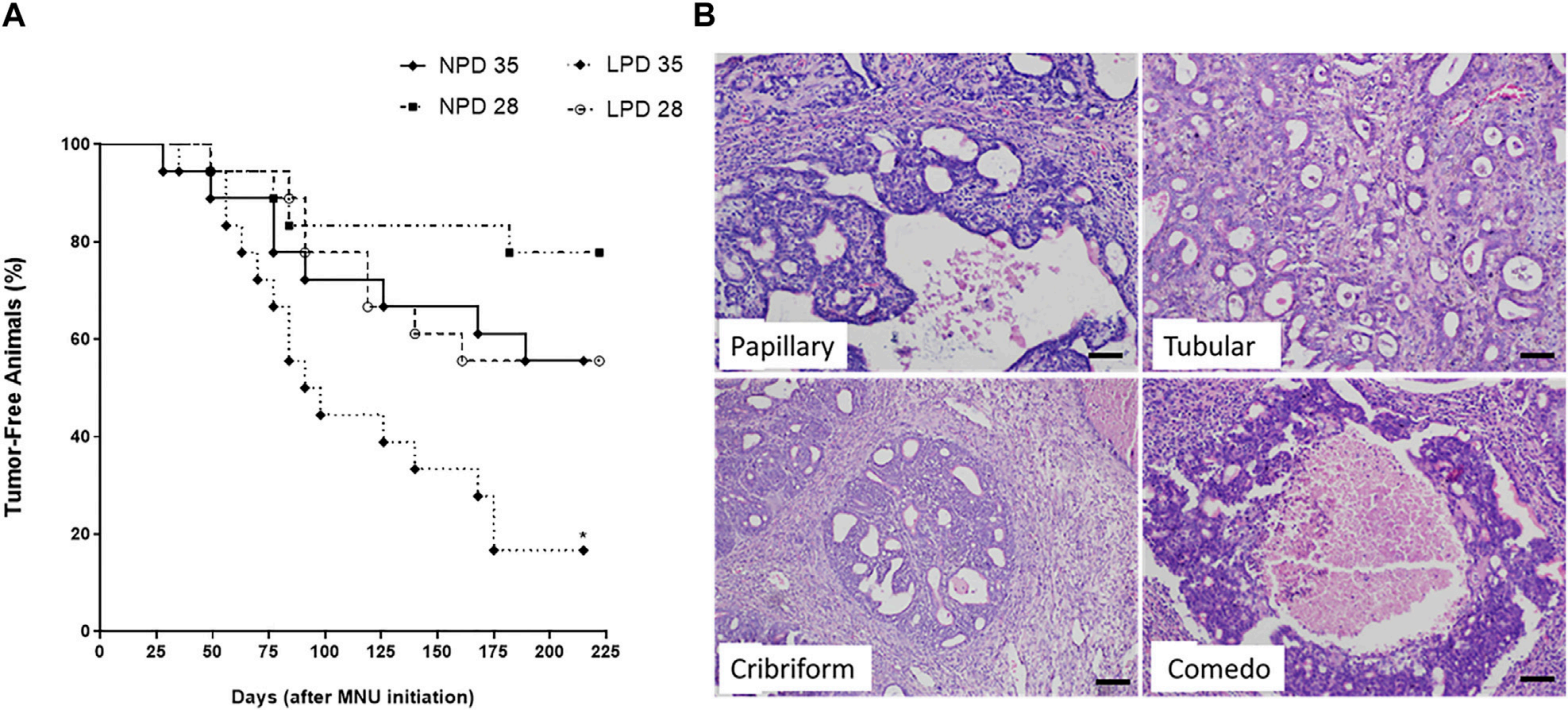
Percentage of tumor-free animals **(A)**. Time represents the MNU-postinitiation days. NPD, normoprotein diet; LPD, low-protein diet. Postnatal day of MNU administration (28 or 35). MNU, *N*-methyl-*N*-nitrosourea administration (25 mg/kg, i.p.; single dose). *Statistically different from NPD 35 (*p* = 0.020). **(B)** Photomicrographs from tumors histological sections stained by hematoxylin–eosin (scale bar = 20 µm).

Considering that maternal low protein intake induces molecular changes in the mammary gland and is also associated with higher chemically induced breast cancer risk in adulthood (Fernandez-Twinn et al., 2007; Zheng et al., 2012), the modifying effects of maternal protein restriction on mammary cancer susceptibility were further investigated through two different timepoints after acute MNU administration (PND 28 or PND 35). Tumor data and percentage of tumor-free animals are shown in Table 2 and Figure 4, respectively. Representative mammary tumor sections are presented in Figure 4. Tumor latency was similar in the LPD groups when compared with their respective NPD groups (*p* = 0.748 and *p* = 0.973 for LPD 28 and LPD 35, respectively). Although not significant (*p* = 1.000), the tumor incidence at the end of experimental period was higher in LPD 28 than in NPD 28 (44% vs. 17%), as well as in LPD 35 in comparison to NPD 35 (84% vs. 44%) (Table 2). However, during MNU-postinitiation days 35–175, the percentage of tumor-free rats of the LPD 35 group fell from 100% to 16%, whereas 56% of the NPD 35 group remained tumor-free (*p* = 0.020) (Figure 4). After histological analysis, the mammary adenocarcinomas were classified as papillary, tubular, comedo, and cribriform subtypes. The most MNU-induced adenocarcinoma showed a papillary pattern in the LPD 28 group (56%), papillary and comedo patterns in the NPD 28 group (50%), tubular pattern in the LPD 35 group (62%), and cribriform pattern in the NPD 35 group (67%) (Table 2 and Figure 4).

**TABLE 2:** Effects of maternal low-protein diet at gestation and lactation on MNU-induced mammary tumors in female offspring.

Parameters	Group/Treatment^a^			
	NPD 28	LPD 28	NPD 35	LPD 35
Number of rats	18	18	18	18
Rat bearing tumor (%)	4/18 (22%)	8/18 (44%)	8/18 (44%)	15/18 (84%)
Tumor latency (days after MNU)^b^	98.00 ± 58.00	106.75 ± 35.2	100.63 ± 56.20	99.87 ± 46.00
Total number of tumors	4	9	9	18
Histological types				
Tubular	0/4	0/9	0/9	11/18 (62%)
Papillary	2/4 (50%)	5/9 (56%)	2/9 (22%)	1/18 (5%)
Cribriform	0/4	1/9 (11%)	6/9 (67%)	5/18 (28%)
Comedo	2/4 (50%)	3/9 (33%)	1/9 (11%)	1/18 (5%)

a NPD, normoprotein diet; LPD, low-protein diet; MNU, *N*-methyl-*N*-nitrosourea administered as a single intraperitoneal dose of 25 mg/kg at postnatal day 28 or 35; PND, postnatal day.

b Values are mean ± standard deviation.

### Maternal Low Protein Intake Does Not Affect Cell Proliferation and Apoptosis But Increases ER-α Expression

**FIGURE 5:**
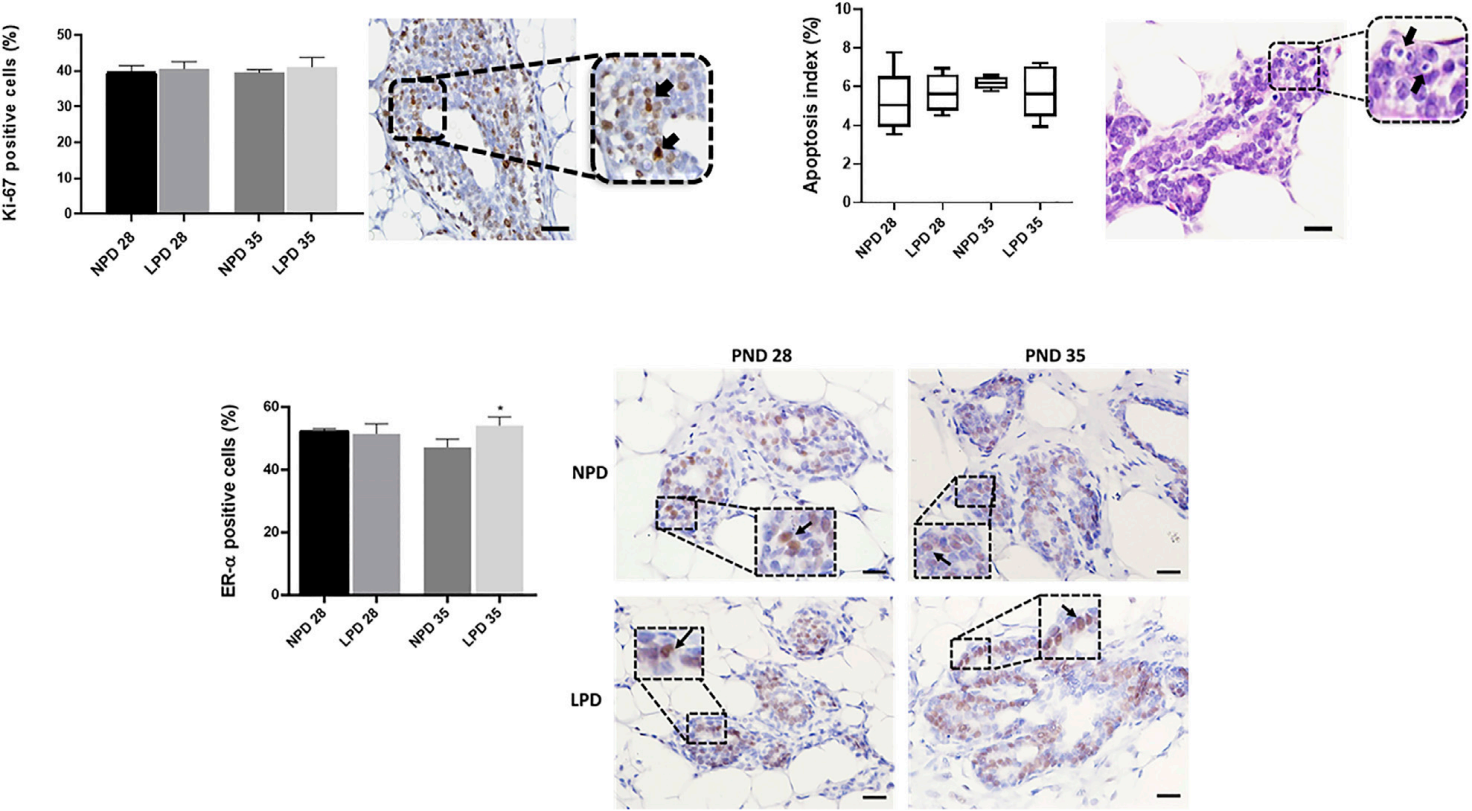
Analysis of cell proliferation, apoptosis, and ER-α in mammary epithelial cells. Labeling index (%) and representative photomicrographs of positive mammary epithelial cells for Ki-67, apoptosis, and ER-α (black arrows) (scale bar = 20 µm). Ki-67 and ER-α values are expressed as mean ± standard deviation and apoptosis values are median and interquartile range (25%–75%). The *p* values to Ki-67 and ER-α were obtained by Student *t* test and *post hoc* Mann–Whitney rank test to apoptosis values. *Significant different from NPD35(*p* ≤ 0.007). NPD, normoprotein diet; LPD, low-protein diet. Postnatal day of MNU administration (28 or 35). MNU, *N*-methyl-*N*-nitrosourea administration (25 mg/kg, i.p.; single dose).

As we observed a “catch-up” mammary growth and a higher mammary tumor incidence in LPD offspring, we tested whether this phenotype was due to a change in the balance between cell proliferation and apoptosis besides the ER-α expression in mammary tissue. Cell proliferation index (Ki-67 staining) and AI were similar between the LPD and NPD groups in both timepoints (*p* > 0.05) (Figure 5). However, the ER-α expression in the offspring mammary epithelial tissue from the LPD 35 group was significantly higher (*p* = 0.007) than in mammary tissue of the NPD 35 group (Figure 5). Thus, this increase in ER-α expression may have contributed to higher tumor susceptibility in adulthood.

### Maternal Low Protein Intake Alters Gene Expression in Mammary Gland After MNU Administration

Given that maternal low protein intake induces genetic changes and the higher mammary tumor incidence observed in LPD 35 female offspring, we assessed 96 genes involved in cell proliferation, DNA damage, DNA repair, and apoptosis by TAC-based real-time PCR. Twenty-four hours after MNU administration, gene expression analysis reported eight differently expressed genes between LPD 28 and NPD 28 offspring and 21 differentially expressed genes between LPD 35 and NPD 35 offspring (Tables 3, 4, Supplementary Data S1). Of the 21 genes, 20 were downregulated and one was upregulated in the mammary tissue of LDP 35 offspring, and of the eight genes, six were upregulated and two were downregulated in the mammary tissue of LPD 28 offspring. Functional enrichment analysis demonstrated that differentially expressed genes in the LPD 35 group belong to 12 functional categories involved in several carcinogenesis-related functions, such as the regulation of cell cycle, G1/S transition of the mitotic cell cycle, apoptotic process, regulation of the apoptotic process, and response to the drug (Table 5). Moreover, these genes enriched 13 molecular pathways, such as microRNAs in cancer, p53 signaling pathway, cell cycle, DNA replication, base excision repair (BER), nucleotide excision repair (NER), and pathways in cancer (Table 6). Among these genes, *Fen1*, *Pold*, *Pole*, and *Ercc2*, which play a crucial role in DNA replication and repair, were all downregulated in LPD 35 (Table 4).

**TABLE 3:** Effects of gestational and lactational low-protein diet on gene expression in female offspring mammary gland after a single MNU administration at PND 28^a^.

Gene symbol	Gene name	Fold change	*p* value
Upregulated genes			
*Aven*	Apoptosis, caspase activation inhibitor	1.707	0.029
*Cd40*	CD40 molecule, TNF receptor superfamily member 5	1.785	0.032
*Ercc1*	Excision repair cross-complementing rodent repair deficiency, complementation group 1	1.510	0.000
Downregulated genes			
*Egfr*	Epidermal growth factor receptor	−1.812	0.045

a Relative expression levels were determined by normalization to *Actb*, *Pum1*, and *Trfc*, 24 h after carcinogen administration. Experimental groups were compared using Student *t* test. Fold change boundary of 1.5 and a *p* value of <0.05 were used. MNU = *N*-methyl-*N*-nitrosourea administered as a single intraperitoneal dose of 25 mg/kg at postnatal day 28 or 35. PND = Postnatal day.

**TABLE 4:** Effects of gestational and lactational low-protein diet on gene expression in female offspring mammary gland after acute MNU administration at PND 35^a^.

Gene symbol	Gene name	Fold change	*p* value
Upregulated genes			
*Cidea*	Cell death–inducing DFFA-like effector a	2.194	0.045
Downregulated genes			
*Api5*	Apoptosis inhibitor 5	−1.522	0.004
*Apaf1*	Apoptotic peptidase activating factor 1	−1.656	0.000
*Atxn3*	Ataxin 3	−2.160	0.000
*Bax*	Bcl2-associated X protein	−2.160	0.000
*Ccnd1*	Cyclin D1	−2.463	0.007
*Ccne1*	Cyclin E1	−2.597	0.002
*Cd44*	Cd44 molecule	−2.469	0.048
*Cdc25a*	Cell division cycle 25 homolog A (S. pombe)	−1.669	0.007
*Dnmt1*	DNA (cytosine-5-)-methyltransferase 1	−2.188	0.007
*Egr1*	Early growth response 1	−2.857	0.015
*Ercc2*	Excision repair cross-complementing rodent repair deficiency, complementation group 2	−1.773	0.002
*Fen1*	Flap structure–specific endonuclease 1	−1.669	0.044
*Foxo3*	Forkhead box O3	−1.527	0.044
*Jun*	Jun oncogene	−2.101	0.003
*Map2k7*	Mitogen activated protein kinase kinase 7	−1.869	0.007
*Mapk8ip1*	Mitogen-activated protein kinase 8 interacting protein 1	−3.546	0.000
*Pold1*	Polymerase (DNA directed), delta 1, catalytic subunit	−2.667	0.005
*Pole*	Polymerase (DNA directed), epsilon	−2.262	0.023
*Prc1*	Protein regulator of cytokinesis 1	−5.495	0.035
*Skp2*	S-phase kinase-associated protein 2 (p45)	−3.597	0.001

a Relative expression levels were determined by normalization to *Actb*, *Pum1*, and *Trfc*, 24 after a single carcinogen administration. Experimental groups were compared using Student *t* test. Fold change boundary of 1.5 and *p *< 0.05 were used. MNU, *N*-methyl-*N*-nitrosourea administered as a single intraperitoneal dose of 25 mg/kg at postnatal day 28 or 35; PND, postnatal day.

**TABLE 5:** Enriched biological process by differential genes expressed in the female offspring mammary gland of LPD 35 group.

	Terms	Gene name	*p* value	Fold enrichment	FDR
GO:0000082	G1/S transition of mitotic cell cycle	*Ccnd1*, *Ccne1*, *Skp2*, *Pole*, *Cdc25a*	5.38E-07	70.76	2.32E-04
GO:0043525	Positive regulation of neuron apoptotic process	*Egr1*, *Jun*, *Bax*, *Foxo3*, *Map2k7*	1.42E-06	55.67	3.06E-04
GO:0051726	Regulation of cell cycle	*Jun*, *Ccnd1*, *Ccne1*, *Bax*, *Skp2*	9.60E-06	34.50	0.0014
GO:0042493	Response to drug	*Egr1*, *Jun*, *Dnmt1*, *Ccnd1*, *Ccne1*, *Bax*, *Foxo3*	1.96E-05	11.07	0.0019
GO:0007568	Aging	*Jun*, *Dnmt1*, *Apaf1*, *Ercc2*, *Bax*, *Foxo3*	2.25E-05	15.90	0.0019
GO:0006915	Apoptotic process	*Ercc2*, *Cidea*, *Bax*, *Foxo3*, *Map2k7*	6.94E-04	11.41	0.0485
GO:0042981	Regulation of apoptotic process	*Egr1*, *Apaf1*, *Cidea*, *Skp2*	9.04E-04	19.65	0.0485
GO:0071310	Cellular response to organic substance	*Egr1*, *Ccnd1*, *Bax*	9.87E-04	61.10	0.0485
GO:0051412	Response to corticosterone	*Ccnd1*, *Ccne1*, *Bax*	0.0011	58.26	0.0485
GO:0000122	Negative regulation of transcription from RNA polymerase II promoter	*Egr1*, *Jun*, *Dnmt1*, *Ccnd1*, *Ccne1*, *Foxo3*	0.0013	6.72	0.0485
GO:0045471	Response to ethanol	*Egr1*, *Dnmt1*, *Ccnd1*, *Ccne1*	0.0013	17.31	0.0485
GO:0034644	Cellular response to UV	*Pold1*, *Bax*, *Cdc25a*	0.0014	52.19	0.0485

Enrichment analysis by DAVID (Database for Annotation, Visualization and Integrated Discovery). FDR, false discovery rate; LPD, group with maternal low-protein diet and *N*-methyl-*N*-nitrosourea administered as a single intraperitoneal dose of 25 mg/kg at postnatal 35.

**TABLE 6:** Enriched molecular pathways by differentially genes expressed in the female offspring mammary gland of LPD 35 group.

KEGG_Pathway	Terms	Gene name	*p* value	Fold enrichment	FDR
rno05161	Hepatitis B	*Jun*, *Apaf1*, *Ccnd1*, *Ccne1*, *Bax*	1.97E-04	15.49	0.007
rno05206	MicroRNAs in cancer	*Dnmt1*, *Ccnd1*, *Ccne1*, *Cdc25a*, *Cd44*	2.20E-04	15.05	0.007
rno05166	HTLV-I infection	*Egr1*, *Jun*, *Ccnd1*, *Pold1*, *Bax*, *Pole*	2.98E-04	8.94	0.007
rno04115	p53 signaling pathway	*Apaf1*, *Ccnd1*, *Ccne1*, *Bax*	4.38E-04	24.60	0.008
rno05222	Small cell lung cancer	*Apaf1*, *Ccnd1*, *Ccne1*, *Skp2*	8.30E-04	19.79	0.012
rno05203	Viral carcinogenesis	*Jun*, *Ccnd1*, *Ccne1*, *Bax*, *Skp2*	0.001	9.16	0.018
rno04722	Neurotrophin signaling pathway	*Jun*, *Bax*, *Foxo3*, *Map2k7*	0.002	13.67	0.021
rno04110	Cell cycle	*Ccnd1*, *Ccne1*, *Skp2*, *Cdc25a*	0.002	13.56	0.021
rno03030	DNA replication	*Fen1*, *Pold1*, *Pole*	0.003	35.88	0.021
rno03410	Base excision repair	*Fen1*, *Pold1*, *Pole*	0.003	34.91	0.021
rno05169	Epstein–Barr virus infection	*Jun*, *Skp2*, *Map2k7*, *Cd44*	0.004	11.64	0.026
rno03420	Nucleotide excision repair	*Pold1*, *Ercc2*, *Pole*	0.005	27.48	0.028
rno05210	Colorectal cancer	*Jun*, *Ccnd1*, *Bax*	0.008	20.18	0.048
rno05200	Pathways in cancer	*Jun*, *Ccnd1*, *Ccne1*, *Bax, Skp2*	0.009	5.44	0.050

Functional enrichment analysis by DAVID (Database for Annotation, Visualization and Integrated Discovery). FDR, False Discovery Rate. LPD, group with maternal low-protein diet and *N*-methyl-*N*-nitrosourea administered as a single intraperitoneal dose of 25 mg/kg at postnatal 35.

The integrated PPI network of LPD 35 deregulated genes shows a higher number of interactions between proteins of the DNA repair, DNA replication, apoptotic process, and cell cycle control (Supplementary Figure S3).

### Maternal LPD Increases DNA Damage in Mammary Epithelial Cells

**FIGURE 6:**
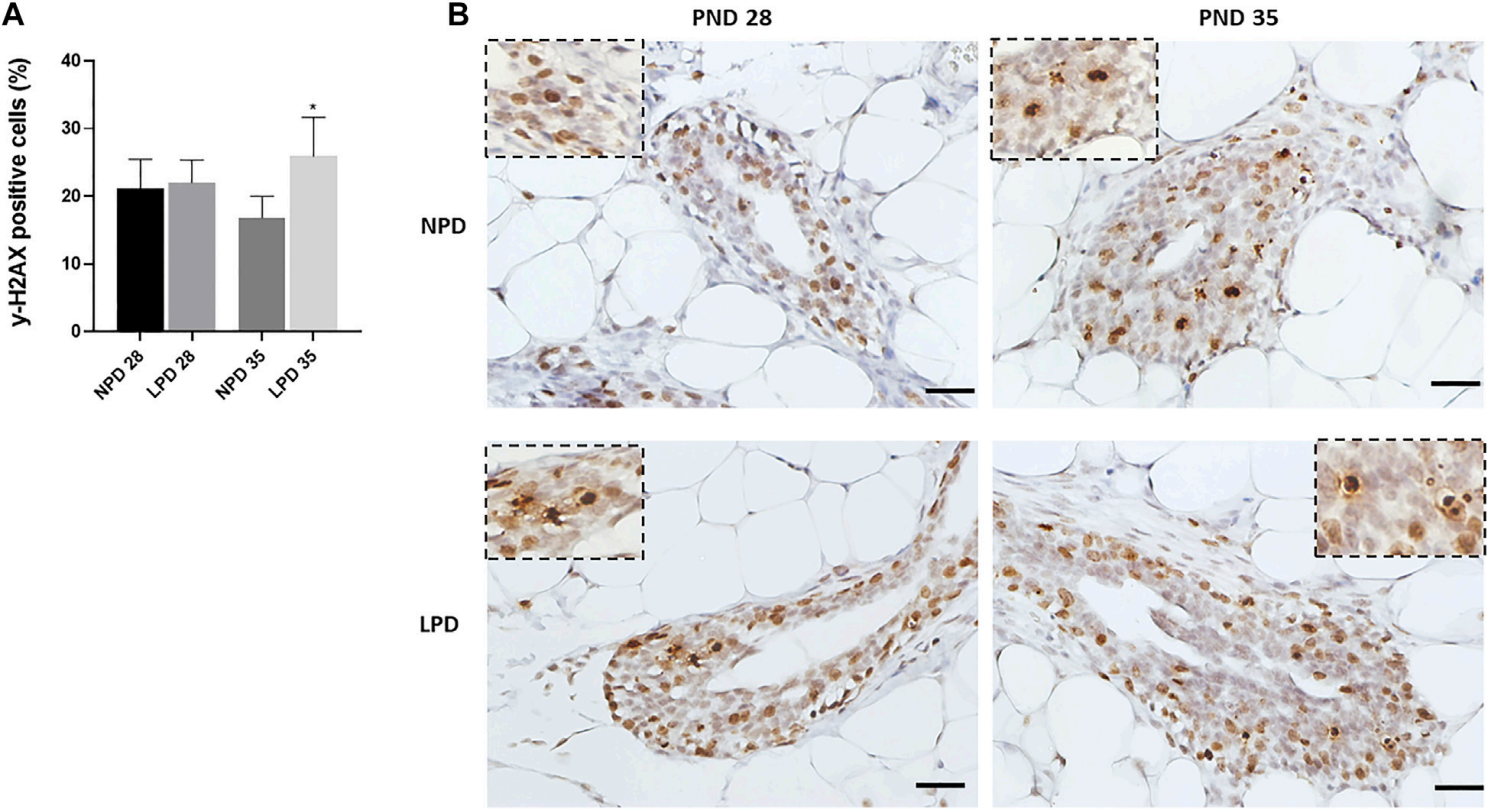
Maternal low-protein diet increase MNU-induced DNA damage in offspring mammary epithelial cells (γ-H2AX). **(A)** γ-H2AX labeling index (%). **(B)** Representative photomicrographs of γ-H2AX-immunostained (scale bar = 20 µm). Values are expressed as mean ± standard deviation. *Statistically different from NPD 35. The *p* values (*p* ≤ 0.042) were determined by Student *t* test. NPD, normoprotein diet; LPD, low-protein diet; PND, postnatal day of MNU administration (28 or 35). MNU, *N*-methyl-*N*-nitrosourea administration (25 mg/kg, i.p.; single dose).

As maternal low protein intake decreased gene expression related to DNA repair, we hypothesized that DNA damage could be higher in female offspring mammary epithelial cells whose dams were fed an LPD in relation to the counterparts whose dams were fed an NPD. Then, the immunoreactivity for phosphorylated (Ser-139 residue) histone H2A.X was evaluated in the mammary gland epithelial cells. The induction of γ-H2AX is one of the earliest events detected in cells after induction of a double-stranded DNA break and provides a sensitive, efficient, and reproducible measurement of the amount of DNA damage (Sedelnikova and Bonner, 2006). The γ-H2AX–positive cells were significantly higher in epithelial mammary cells of LPD 35 when compared with the NPD 35 group (*p* = 0.042), 24 h after a single dose of MNU administration (Figure 6). Thus, a higher DNA damage level and a reduced DNA repair capacity could have contributed to mammary carcinogenesis susceptibility increased in LPD 35 offspring.

### Prognostic Value of Deregulated Genes in Female Offspring Mammary Gland by Maternal LPD

The set of differentially expressed mammary genes in the LPD 35 group was further chosen for a translational approach involving the species-comparative *in silico* analysis on the basis of the human BRCA-TCGA invasive carcinoma dataset of patients with breast cancer including survival risk assessment. Using the 21 deregulated genes found in the LPD 35 group in comparison to the NPD 35 group, the univariate Cox analysis selected 11 genes for breast cancer prognostic prediction (*p* < 0.1). These genes were further used in multivariate Cox analysis when a significant *p* value was considered as 0.05. We identified that the high expression of *Cidea* gene predicts a lower risk of survival for patients with breast cancer (hazard ratio = 1.511 and *p* = 0.001) (Table 7). Thus, these results suggest that maternal low protein intake deregulates gene expression in female offspring mammary glands, which are associated with poor prognostic prediction of patients with breast cancer.

**TABLE 7:** Hazard ratio and confidence intervals of human genes overlapping with differentially expressed genes in LPD 35 rat mammary tissue.

Univariate analysis			
Genes	Hazard ratio	Confidence interval (95%)	*p* value
*Cidea*	1.600	1.356–1.889	2.82E-08
*Fen1*	0.386	0.241–0.621	0.000
*Prc1*	0.545	0.410–0.723	0.000
*Cdc25a*	0.686	0.533–0.883	0.003
*Dnmt1*	0.330	0.159–0.687	0.003
*Pold1*	0.559	0.371–0.842	0.005
*Bax*	0.475	0.273–0.830	0.009
*Egr1*	1.447	1.090–1.922	0.011
*Pole*	0.385	0.182–0.812	0.012
*Foxo3*	2.570	1.194–5.532	0.016
*Ccnd1*	0.721	0.503–1.034	0.076
*Ercc2*	0.554	0.258–1.190	0.130
*Jun*	1.360	0.856–2.161	0.193
*Api5*	0.423	0.115–1.548	0.194
*Ccne1*	0.896	0.712–1.129	0.352
*Cd44*	0.781	0.445–1.369	0.388
*Map2k7*	1.504	0.571–3.959	0.409
*Atxn3*	1.363	0.557–3.334	0.497
*Mapk8ip1*	1.104	0.782–1.558	0.573
*Skp2*	0.927	0.671–1.280	0.644
*Apaf1*	1.084	0.530–2.216	0.826
Multivariate analysis			
Genes	Hazard ratio	Confidence interval (95%)	*p* value
*Cidea*	1.5111	1.192–1.916	0.001
*Foxo3*	2.101	0.830–5.317	0.117
*Egr1*	0.787	0.529–1.171	0.238
*Fen1*	0.5636	0.197–1.616	0.286
*Ccnd1*	0.7942	0.505–1.250	0.319
*Bax*	1.4183	0.546–3.683	0.473
*Cdc25a*	1.1467	0.672–1.957	0.616
*Prc1*	0.8626	0.454–1.640	0.652
*Pold1*	0.8308	0.344–2.007	0.680
*Dnmt1*	1.0813	0.318–3.677	0.900
*Pole*	1.0113	0.346–2.957	0.984

Biomarker comparison and validation of Survival gene expression data by ServExpress software. Dataset: BRCA-TCGA breast-invasive carcinoma. Hazard ratio was estimated by fitting a CoxPH using risk group as covariate. LPD, group with maternal low-protein diet and *N*-methyl-*N*-nitrosourea administered as a single intraperitoneal dose of 25 mg/kg at postnatal 35.

## Discussion

The molecular mechanisms associated with increased cancer susceptibility by maternal protein restriction using chemically induced mammary carcinogenesis models are still poorly understood. In this study, we evaluated the deleterious effects of gestational and lactational low protein intake on susceptibility to MNU-induced mammary carcinogenesis in female offspring rats, as well as on the gene expression of key pathways involved in mammary tumor initiation. Our data suggest that maternal low protein intake plays a role in programming the female offspring mammary cancer susceptibility through an increase in DNA damage and deregulation of DNA repair and DNA replication pathways after a single dose of carcinogen MNU in female SD offspring.

Maternal postconception and paternal preconception protein restriction have been associated with an increased susceptibility to early-onset chemically induced mammary tumorigenesis in the female offspring (Fernandez-Twinn et al., 2007; da Cruz et al., 2018). In the present study, even though the tumor incidence at the end of experimental period was similar between female offspring from the NPD and LPD groups, the number of tumor-free rats was significantly higher in the NPD 35 group when compared with the LPD 35 group. These results show that a maternal low protein intake increased the susceptibility to chemically induced mammary cancer in adulthood, which can be explained by morphological and molecular alterations associated with a differential response to MNU tumor initiation observed in this model. In addition to the deleterious effects of maternal protein restriction, previous studies have also shown that dietary fats are associated with an increased risk of breast cancer for mothers and female offspring (de Assis et al., 2012; de Oliveira Andrade et al., 2014; Engin, 2017; Grassi et al., 2019). Maternal high caloric intake of dietary fats/sugar increases serum estrogens during pregnancy and induces expansion of the mammary stem cell compartment during mammary development, increasing breast cancer risk in female offspring (de Assis et al., 2012; Lambertz et al., 2017).

According to the “thrifty phenotype” hypothesis, fetal malnutrition may induce physiological and/or metabolic adaptations to ensure nutrient supply to the most vital organs (such as the brain) at the expense of other organs (Hales and Barker, 2001). Studies have demonstrated that protein restriction at gestational and lactational phases impairs mammary gland development, and a compensatory growth can be observed after an adequate-protein diet supply (Fernandez-Twinn et al., 2007; Beinder et al., 2014). In the present study, the mammary gland development was also impaired in the LPD groups followed by a “catch-up” growth after an adequate-protein diet supply. Furthermore, the total number of the TEBs was reduced in LPD 21 and LPD 28 groups, which is in line with the mammary impairment and compensatory growth observed.

The mammary gland development is sensitive to steroid hormones, and an increased expression of progesterone, estrogen, and estrogen receptors has been detected in the mammary gland during the catch-up mammary growth phase after adequate-protein diet introduction (Cavalieri et al., 2006; Fernandez-Twinn et al., 2007; Arendt and Kuperwasser, 2015; Russo, 2015). Estrogen and progesterone serum levels were similar between female offspring from the NPD and LPD groups. However, the ER-α expression in epithelial mammary tissue was significantly higher in the mammary gland from the LPD 35 group when compared with the NPD 35 group. Therefore, it is possible that the high mammary epithelial ER-α expression could have influenced the lower tumor-free animal in the LPD 35 group.

The increased susceptibility to chemically induced mammary cancer in female offspring from the LPD 35 group at adulthood may also be explained by molecular changes observed after acute response induced by MNU administration. DNA is continually exposed to endogenous and exogenous damaging agents, and failure in cell cycle regulators and DNA repair pathways drive tumor initiation (Khanna, 2015; Jeggo et al., 2016; Roos et al., 2016). In our analysis, a single MNU dose at PND 35 resulted in 21 differentially regulated genes in the LPD group. These genes belong to functional categories involved in cell cycle regulation, G1/S transition of the mitotic cell cycle, apoptotic process, and acute response to the carcinogen. Moreover, the deregulated genes also enriched some pathways such as microRNAs in cancer, p53 signaling pathway, cell cycle, DNA replication, BER, NER, and pathways in cancer. The molecular mechanisms involved in chemically induced mammary carcinogenesis susceptibility by maternal low protein intake are still poorly understood. In absence of carcinogen administration, the most molecular findings in mammary gland from female offspring with maternal protein restriction addressed transcriptional alterations toward cell cycle control, insulin resistance, and ROS pathways (Zheng et al., 2012; Beinder et al., 2014). Furthermore, these studies evaluated the effects of LPD only in gestation phase; meanwhile, the most observed scenario in population is a poor protein diet during gestation and lactation. Therefore, our gene expression profile is innovative in showing different altered pathways in female offspring mammary gland after a gestational and lactational low protein scenario and MNU exposure.

During the G1 phase of cell cycle, several metabolic, stress, and environmental signals influence the G1/S transition and cell division stop (Morgan, 2007; Rhind and Russell, 2012). Besides, to enter S phase, the cyclin-dependent kinase must be activated, and one of the mechanisms to keep Cdk2 inactive is based on limiting the supply of cyclin E (Rhind and Russell, 2012). In the present study, the *Ccnd1*, *Ccne1*, *Skp2*, and *Jun* gene expression was lower in mammary tissue from the LPD 35 group when compared with the NPD 35 group. These genes are required for cell cycle progression and for regulating the G1/S and G2/M phases transition (Wang et al., 2000; Gstaiger et al., 2001; Nakayama et al., 2010; Xu and Lin, 2018). Therefore, the low expression of these genes may be an attempt to stop cell division and avoid genomic instability. Furthermore, the p53 pathway was enriched by downregulation of *Ccne1*, *Ccnd1*, *Apaf-1*, and *Bax* genes in the LPD 35 group when compared with the NPD 35 group. The p53 pathway blocks cyclin D and cyclin E leading to cell cycle arrest and also induces apoptosis through many genes, such as *Bax* and *Apaf-1* (Harris and Levine, 2005; Joerger and Fersht, 2016; Cheok and Lane, 2017). The *Egr1* gene was also downregulated in the LPD 35 group and targets the *Pten* promoter, resulting in tumor cell apoptosis (Chen et al., 2010; Wang et al., 2021). Furthermore, *Egr1* acts on the TP53 promoter and initiates the expression of p53, which in turn activates *Egr1* forming a feedback loop (Yu et al., 2007; Wang et al., 2021). Based on these findings and considering that the AI was not different in the LPD groups, the p53 pathway may have contributed to cell cycle arrest inducing *Ccne1* and *Ccnd1* downregulation, but it was not directed toward apoptosis in response to DNA damage. On the other hand, the downregulation of *Fen1*, *Pole*, and *Pold1* genes may have impaired DNA replication accuracy and overlapped the cellular response of stopping the cell cycle (Agbor et al., 2013; Tsutakawa et al., 2014; Nicolas et al., 2016). As a consequence, impaired DNA replication accuracy could contribute to the high mammary carcinogenesis susceptibility observed in the LPD 35 group compared with the NPD 35 group.

Beyond the downregulation of *Fen1*, *Pold1*, and *Pole*, we also observed a reduction in the expression of *Ercc2* gene in the offspring mammary tissue of LPD 35 compared with NPD 35. These genes act in the cellular response to DNA damage where *Fen1* participates in the BER pathway, the *Ercc2* in the NER pathway, and the *Pole* and *Pold1* in both pathways (Balakrishnan and Bambara, 2013; Nicolas et al., 2016; Mouw et al., 2017). Therefore, these functional pathways might have impaired the DNA damage repair and contributed to the tumor initiation of mammary epithelial cells in our LPD 35 group.

Based on gene expression findings, we hypothesized that maternal protein restriction increased the DNA damage after a single dose of MNU in the female offspring mammary epithelial cells. The H2AX protein is a variant of histone H2A and, after induction of double-stranded DNA breaks, becomes phosphorylated to form gamma-H2AX (γ-H2AX). The induction of γ-H2AX is one of the earliest events detected in cells following exposure to DNA damaging agents and provides a sensitive, efficient, and reproducible measurement of the amount of DNA damage (Sedelnikova and Bonner, 2006; Mah et al., 2010). Through immunohistochemistry, we observed a higher LI for γ-H2AX epithelial cells in the mammary gland from the LPD 35 group compared with the NPD 35 group. Genomic instability is a hallmark of tumors, and mutations can arise due to increased DNA damage exposure and/or decreased DNA repair capacity (Negrini et al., 2010; Ferguson et al., 2015). Therefore, our data allow us to suggest that maternal LPD increases the DNA damage directly or/and through deregulation of DNA repair and DNA replication in the female offspring mammary epithelial cells, 24 h after MNU insult, especially at PND 35. Finally, these transcriptional and functional postnatal events resulted in increased mammary tumor susceptibility in female offspring at adulthood.

When DNA damage is not repaired, programmed cell death or apoptosis is activated to eliminate cells with extensive gene instability (Chae et al., 2016). The expression of *Apaf1*, *Bax, Egr1*, *Skp2*, *Foxo3*, *Map2k7*, and *Ercc2* genes was significantly lower in the mammary tissue from the LPD 35 group compared with the NPD 35 group. These genes participate in the positive regulation of the apoptotic process. In mammalians, the Foxo subfamily includes four genes of forkhead box-O transcription factors (Foxos), Foxo1, Foxo3, Foxo4, and Foxo6 that play a key role in cancer (Weidinger et al., 2008). Especially in humans, *Foxo*3a mediates a variety of cellular processes including apoptosis, proliferation, cell cycle progression, DNA damage, and tumorigenesis (Liu et al., 2018). In addition, the loss of *Foxo*3a expression predicted poor prognosis in human breast cancer, probably by regulating breast cancer stem cell properties (Liu et al., 2020). Thus, considering the differential gene expression of *Foxo3* and other genes (i.e., *Apaf1*, *Bax*, *Egr1*, *Skp2*, and *Ercc2*), the negative regulation of the apoptotic process could be one of the mechanisms that led to an increased susceptibility to chemically induced mammary carcinogenesis in the LPD 35 group. However, when performing the morphological analysis to detect cells in apoptosis, there was no difference in the apoptotic index between the NPD and LPD groups. Thus, these molecular alterations could have contributed to apoptosis induction in a later postnatal phase, but without a predilection for epithelial cells initiated by MNU, resulting in higher risk for mammary carcinogenesis. As gene expression analysis was detected in whole mammary tissue (epithelium and stroma), whereas γ-H2AX, Ki-67 and apoptosis was analyzed only in the epithelial tissue, it can be considered as a limitation in this study.

The expression of *Cidea* gene has been correlated with apoptosis induction in different types of tumors, such as breast cancer (Silva et al., 2014; Bortololto et al., 2014), and is directly proportional to DNA fragmentation (Omae at al., 2012; Bortolo et al., 2017). As discussed, when the DNA damage is not repaired, programmed cell death or apoptosis is activated. When genes and proteins that positively control apoptosis are highly expressed in tumors, it seems favorable to prognosis due to the capacity for apoptosis induction. On the other hand, the increase in these genes also shows a high level of DNA damage. Thus, in some cases, such as in breast cancer, the high expression of these genes is correlated to a poor survival rate. The *in silico* analysis of human breast cancer, the TCGA dataset shows a poor prognosis of patients with *Cidea* upregulation. Similarly, we found this gene upregulated in offspring mammary gland of LPD 35 group, which may suggest that maternal LPD could deregulate genes possibly leading to increased risk of mammary cancer development and/or poor prognosis. In humans, *Cidea* is positively correlated with insulin sensitivity and healthy obesity. However, it is unknown whether Cidea causes the metabolically healthy phenotype (Abreu-Vieira et al., 2015). Even though the LPD female offspring did not develop obesity during postnatal and adult life, *Cidea* gene is linked to development of the metabolic changes and insulin resistance as described by others in this maternal malnutrition model (Zambrano et al., 2005; Fernandez-Twinn et al., 2007). This result highlights the negative impact of maternal LPD on female offspring mammary gland.

As shown in our findings, residual mammary gland growth was still observed at PND 29 (24 h after MNU administration) in the LPD group. Steroid hormones, growth hormone (GH), and prolactin are the master regulators of mammary growth. However, peptide growth factors such as epidermal growth factor (EGF), fibroblast growth factor, and IGFs and their receptors have specific roles during mammary gland development (Kleinberg and Ruan, 2008; Hynes and Watson, 2010). Each stage of mammary gland development has distinct patterns of gene expression and specific hormonal requirements that influence the cross-talk between epithelium and mesenchyme to regulate its development (Wiesen et al., 1999; Sternlicht, 2005; Hynes and Watson, 2010). Stromal–epithelial interactions are critical in determining patterns of growth, development, and ductal morphogenesis, and the EGF contributes to these stromal–epithelial interactions (Wiesen et al., 1999; Sternlicht, 2005; Hynes and Watson, 2010). In our study, the expression of epidermal growth factor receptor, a gene that regulates mammary gland ductal outgrowth with proliferative and survival roles (Sternlicht, 2005; Hynes and Watson, 2010), was significantly downregulated in mammary glands from LPD 28 group compared with NPD 28. Thus, the maternal LPD could lead to an impairment of mammary gland development in female offspring through downregulation of *Egfr* gene. The analysis of associated changes in epithelial–mesenchymal cross-talk remains to be addressed in further studies, as the mammary gland was analyzed in whole in our study.

As discussed, morphological mammary changes were observed at PND 28, whereas relevant molecular alterations and significant tumor susceptibility were observed after MNU administration at PND 35. It may be due to the differential mammary window of susceptibility to carcinogen initiation, without an important influence of mammary gland “catch-up” growth phase after NPD reintroduction.

In conclusion, the maternal low protein intake enhances MNU-induced DNA damage and deregulates DNA repair and DNA replication pathways in F1 female offspring mammary gland, which can be associated with an increase in mammary tumor development in female offspring in adulthood. These findings advance the knowledge of early-transcriptional mammary changes programmed by gestational and lactational LPD with long-term effects on mammary carcinogenesis susceptibility. Further epigenetic and proteomic studies are needed to clarify the underlying mechanisms and identify novel biomarkers.

## Data Availability

The original contributions presented in the study are included in the article/Supplementary Material, further inquiries can be directed to the corresponding authors.
