# Supplementary material for: Maternal Low-Protein Diet Deregulates DNA Repair and DNA Replication Pathways in Female Offspring Mammary Gland Leading to Increased Chemically Induced Rat Carcinogenesis in Adulthood
Source: Front Cell Dev Biol. 2022 Feb 1;9:756616. doi: 10.3389/fcell.2021.756616 (PMC8844450; doi:10.3389/fcell.2021.756616)

Supplementary Material

Supplementary Figure 1. Experimental design.

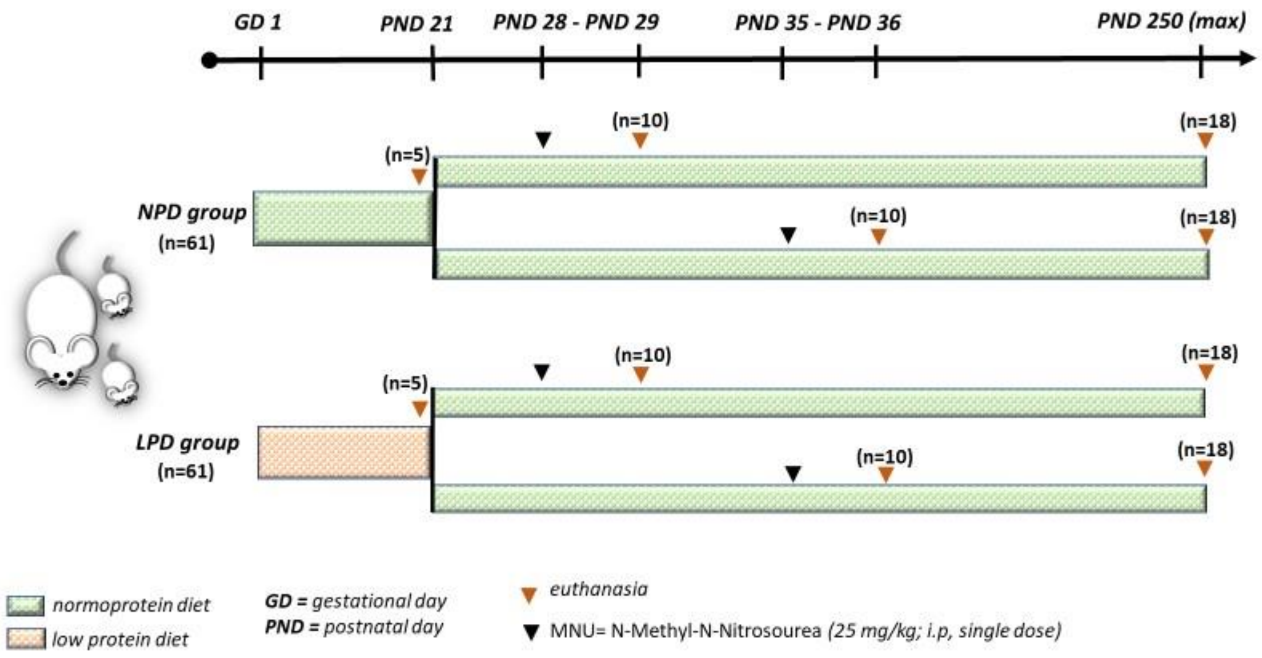

Supplementary Figure 2. Serum 17β-estradiol and progesterone level in female offspring.

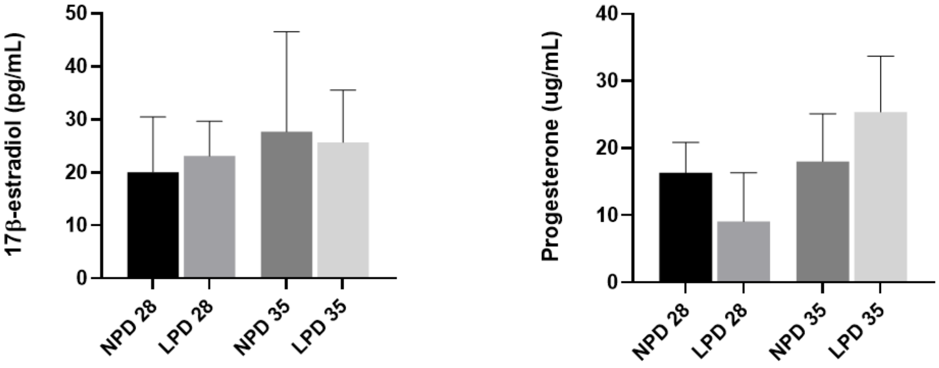

NPD: normoprotein diet. LPD: low protein diet. Postnatal day of MNU administration (28 or 35). MNU= N-methyl-N-nitrosourea administration (25mg/kg; i.p; single dose).

Supplementary Figure 3. Protein–protein interaction (PPI) network in female offspring mammary gland exposed to gestational and lactational low protein and after acute N-methyl-N-nitrosourea administration on postnatal day 35. Lines highlight PPI, with Ccne1, Ccnd1, Cdc25a, Skp2, Pold1, Pole, and Fen1 presenting the strongest and highest number of interactions. STRING v10.5.1 was used to generate protein interactions, and the resulting network was visualized using Cytoscape v3.4.0.

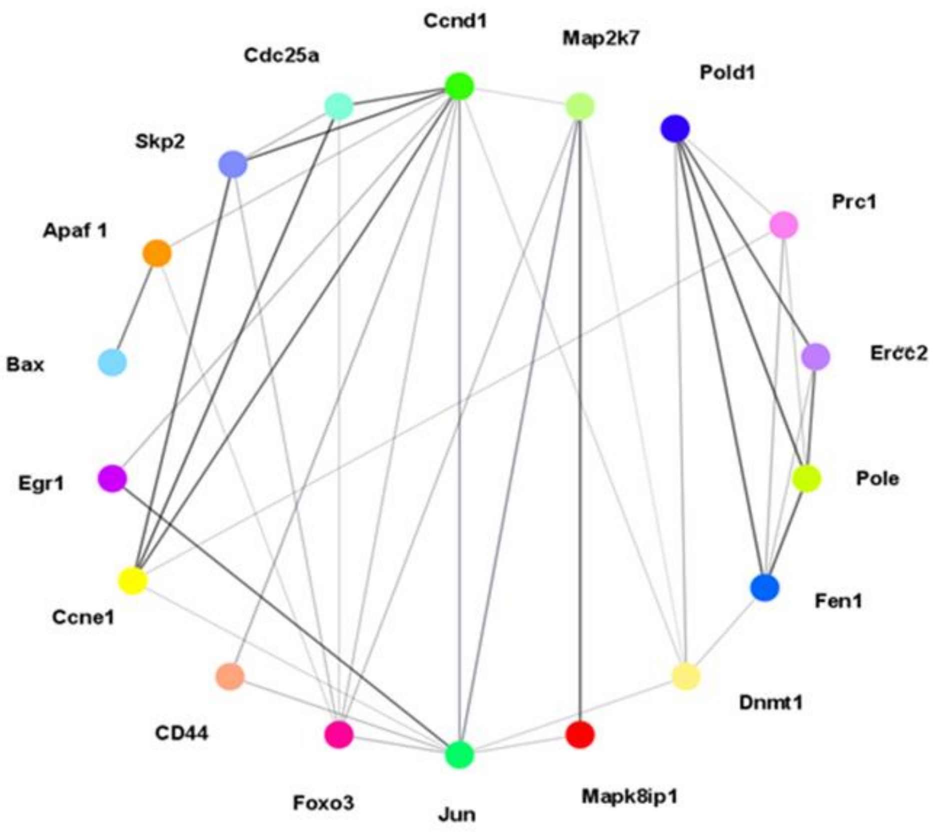

Supplement: Supplementary file 1 [file DataSheet2.pdf]
