# Supplementary material for: Maternal Low-Protein Diet Deregulates DNA Repair and DNA Replication Pathways in Female Offspring Mammary Gland Leading to Increased Chemically Induced Rat Carcinogenesis in Adulthood
Source: Front Cell Dev Biol. 2022 Feb 1;9:756616. doi: 10.3389/fcell.2021.756616 (PMC8844450; doi:10.3389/fcell.2021.756616)
Supplement: Supplementary file 2 [file Table1.docx]

Supplementary Material

.

**Supplementary Table 1.** Normal protein diet (NPD) or low protein diet (LPD) profile (g/kg in weight or amount in weight) from semi-purified diets.*

| **Ingredients** | **NPD (17% protein)** | **LPD (6% protein)** |
| --- | --- | --- |
| Casein (84% of protein) | 202 | 71.5 |
| Amido | 397 | 480 |
| Dextrin | 130.5 | 159 |
| Sucrose | 100 | 121 |
| L-cystine | 3 | 1 |
| Fiber pH 101 ou pH 102 (microcellulose) | 50 | 50 |
| Soy oil | 70 | 70 |
| Salts mix AIN93G* | 35 | 35 |
| Vitamins mix AIN93G* | 10 | 10 |
| Choline Hydrochloride / Bitartrate | 2.5 | 2.5 |
| Energy Intake (Kcal/g^1^) | 3.96 | 3.96 |

* Based in AIN-93G Growth Purified Diet for rodents.
